# Predicting Hemorrhagic Transformation of Acute Ischemic Stroke: Prospective Validation of the HeRS Score

**DOI:** 10.1097/MD.0000000000002430

**Published:** 2016-01-15

**Authors:** Elisabeth B. Marsh, Rafael H. Llinas, Andrea L.C. Schneider, Argye E. Hillis, Erin Lawrence, Peter Dziedzic, Rebecca F. Gottesman

**Affiliations:** From the Johns Hopkins School of Medicine, Department of Neurology (EBM, RHL, AEH, PD, RFG); Johns Hopkins Bayview Medical Center (EBM, RHL, EL, RFG); and Johns Hopkins Bloomberg School of Public Health, Department of Epidemiology, Baltimore, MD, USA (ALCS, RFG).

## Abstract

Hemorrhagic transformation (HT) increases the morbidity and mortality of ischemic stroke. Anticoagulation is often indicated in patients with atrial fibrillation, low ejection fraction, or mechanical valves who are hospitalized with acute stroke, but increases the risk of HT. Risk quantification would be useful. Prior studies have investigated risk of systemic hemorrhage in anticoagulated patients, but none looked specifically at HT. In our previously published work, age, infarct volume, and estimated glomerular filtration rate (eGFR) significantly predicted HT. We created the hemorrhage risk stratification (HeRS) score based on regression coefficients in multivariable modeling and now determine its validity in a prospectively followed inpatient cohort.

A total of 241 consecutive patients presenting to 2 academic stroke centers with acute ischemic stroke and an indication for anticoagulation over a 2.75-year period were included. Neuroimaging was evaluated for infarct volume and HT. Hemorrhages were classified as symptomatic versus asymptomatic, and by severity. HeRS scores were calculated for each patient and compared to actual hemorrhage status using receiver operating curve analysis.

Area under the curve (AUC) comparing predicted odds of hemorrhage (HeRS score) to actual hemorrhage status was 0.701. Serum glucose (*P* < 0.001), white blood cell count (*P* < 0.001), and warfarin use prior to admission (*P* = 0.002) were also associated with HT in the validation cohort. With these variables, AUC improved to 0.854. Anticoagulation did not significantly increase HT; but with higher intensity anticoagulation, hemorrhages were more likely to be symptomatic and more severe.

The HeRS score is a valid predictor of HT in patients with ischemic stroke and indication for anticoagulation.

## INTRODUCTION

Hemorrhagic transformation (HT) increases morbidity and mortality of acute ischemic stroke.^1^ Ischemia leaves the cerebral vasculature friable, resulting in the highest rate of HT in the days immediately following infarction.^2–4^ Anticoagulants both increase risk of HT,^5^ and worsen degree of bleeding.^6^ There are multiple scores that predict systemic hemorrhage in patients taking anticoagulants for atrial fibrillation,^7,8^ but few include intracerebral hemorrhage, and none address HT of ischemic stroke (Table 1). Patients presenting with acute ischemic stroke often require anticoagulation, most commonly for cardiac etiologies such as atrial fibrillation, reduced ejection fraction, or mechanical valves. There is a known daily risk of stroke when anticoagulation is held; however, risk of HT is poorly characterized. Quantified risk stratification would allow for more appropriate monitoring for HT in high risk patients, and provide a tangible assessment of risk when counseling families of potential complications.

**TABLE 1:**
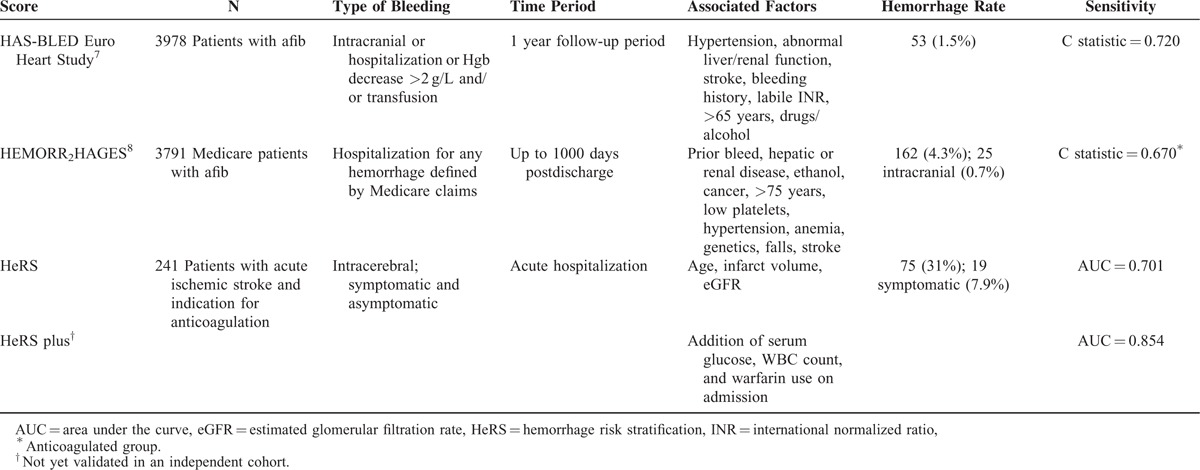
Comparison of the Predictive Models of Hemorrhage

### Factors Predicting Hemorrhage

In a previous study, we evaluated factors associated with increased risk of HT in a cohort of patients hospitalized with acute ischemic stroke who also had an indication for anticoagulation (n = 123).^6^ Demographic information (gender, race, and age); medical factors (blood pressure, glycemic control, lipid profile, and renal function); information pertaining to anticoagulation status and other medications (prehospital use, indication for anticoagulation, anticoagulation status while hospitalized, peak international-normalized ratio (INR) and PTTr values, and days to therapeutic anticoagulation poststroke); and information on stroke size and severity (NIHSS score, infarct volume, and Trial of Org 10172 in Acute Stroke Trial classification) were collected and analyzed. Renal function was calculated on admission using the Modification of Diet in Renal Disease (MDRD) Equation^9^), and defined by both linear estimated glomerular filtration rate (eGFR) and category of impairment: normal (eGFR ≥60 mL/min/1.73 m^2^); mild (eGFR 30–59 mL/min/1.73 m^2^); and moderate (eGFR < 30 mL/min/1.73 m^2^).^6^ All initial and follow-up neuroimaging obtained during the hospitalization was reviewed for infarct volume and evidence of HT. Hemorrhages were classified as: petechiae versus hematoma; and symptomatic versus asymptomatic. In an adjusted model, advanced age (odds ratio [OR] per 10 years 1.31; 95% confidence interval [CI] 0.98–1.74), renal impairment (1.81 per worsening category of eGFR; 95% CI 1.01-3.26), and larger infarct volume (OR 1.13 per 10 cc; 95% CI 1.05–1.21) were significant predictors of HT.^6^ Interestingly, neither the use of anticoagulation during the hospital stay nor time from stroke onset to initiation of heparin, a heparioid, or warfarin were significant predictors of hemorrhage, though use was associated with larger more severe hemorrhages.^6^ Regression coefficients from multivariable logistic regression were used to predict odds of hemorrhage. Probability of hemorrhage was then calculated, creating a hemorrhage risk stratification (HeRS) score. Given the complexity of the calculation, we designed an iPhone application to perform this calculation. Search Apple app store: “Johns Hopkins HeRS.”

In this analysis, we validate the HeRS score in a unique, prospectively followed inpatient cohort with acute ischemic stroke and an indication for anticoagulation.

## METHODS

### Patient Population

This prospective cohort study was approved by the Johns Hopkins School of Medicine's Institutional Review Board. It was observational in nature and performed as part of a quality assurance initiative using de-identified data stored in the Johns Hopkins Bayview Stroke/Hemorrhage Database (NA_00079956). Therefore, informed consent was not required. Adults (18 years of age and older) presenting to the Johns Hopkins Hospital or Johns Hopkins Bayview Medical Center between June 2011 and March 2014 with: an acute ischemic stroke on head computerized tomography (CT) or diffusion-weighted magnetic resonance imaging (MRI), and a known condition typically requiring treatment with anticoagulation ware included in analysis. Patients were excluded from analysis if complete data on infarct volume, age, and eGFR were unavailable (n = 0). Unlike our original cohort, patients were prospectively identified by physicians on our Stroke Service and Neurology Consult Team rather than retrospectively using ICD-9 codes. Indications for anticoagulation included: atrial fibrillation, basilar artery thrombosis, cervical arterial dissection, mechanical valve (aortic or mitral), reduced ejection fraction (≤35%), myocardial infarction, apical thrombus, pulmonary embolus or deep vein thrombosis, hypercoagulable state, and high-risk intracranial/extracranial large-vessel stenosis.^6^ Decisions to anticoagulate were made by the primary team caring for the patient. A patient was defined as “anticoagulated” if they received warfarin, unfractionated/low molecular weight heparin (at treatment doses), or one of the new oral anticoagulants.^6^ In nearly 100% of patients, intravenous heparin was used (dosing based on our institution's unfractionated heparin nomogram: activated partial thromboplastin time [aPTTr] goal 1.5–2.0) as a bridge to warfarin therapy. Potential patients were identified by the inpatient stroke attending physician, and charts were reviewed to determine eligibility. A total of 242 patients were identified over the 2.75-year period. Similar to prior analyses, data were collected regarding patient demographics (age, race, and sex), medical profile (admission blood pressure, history of diabetes, statin use, antiplatelet use, anticoagulation status—agent and timing, baseline laboratory values—including eGFR estimated using the MDRD equation^9^), and stroke characteristics (National Institutes of Health [NIH] Stroke Scale, treatment with tissue plasminogen activator [tPA], and infarct volume). Patients were prospectively followed through hospital discharge for HT, defined as bleeding into the area of infarction. Only those with complete data on age, infarct volume, and eGFR were included in the final analysis (n = 241).

### Hemorrhage Risk Stratification (HeRS) Score Calculation

Using regression coefficients generated through multivariable logistic regression in our prior study,^6^ the odds of hemorrhage was predicted by exponentiating the equation: log(odds) = −3.823563 + (0.0120706) × (Volume) + (0.5939482) × (eGFR Category) + (0.0266442) × (Age). Both age (years) and volume (cc) were expressed as continuous variables. eGFR category was defined as: 0 = eGFR ≥60 mL/min/1.73 m^2^; 1 = eGFR 30–59 mL/min/1.73 m^2^; 2 = eGFR <30 mL/min/1.73 m^2^. The probability of hemorrhage (HeRS score) was then calculated using the equation: HeRS score = odds/(1 + odds).

### Imaging Data

Patients underwent an initial MRI of the brain as part of their standard stroke evaluation. Neuroimaging was reviewed by 2 board-certified vascular neurologists (EBM, RHL) independent from the clinical record. Imaging was performed on a 1.5 or 3.0 Tesla scanner using a standard quadrature transmit-receive head coil. In the majority of cases, MRI was obtained within 24 to 72 hours of admission. Areas of restricted diffusion were identified on diffusion-weighted imaging. Given its ease of calculation, infarct volume was estimated using the validated equation: (length × width × (slice thickness × number of slices))/2.^10^ Patients unable to undergo MRI due to claustrophobia or pacemaker placement (n = 42) had noncontrast CT imaging. Volumes were estimated using the same technique. To determine HT, all follow-up neuroimaging performed during a patient's hospitalization was reviewed for intracerebral bleeding. No patient experienced isolated hemorrhage outside of the ischemic bed. Hemorrhages were classified as symptomatic (defined as any subjective clinical worsening determined by the treating physicians [no specified change in NIH stroke scale required], with blood on corresponding head imaging^11^) versus asymptomatic, and were further classified by severity based on European Cooperative Acute Stroke Study criteria (hemorrhagic infarction [HI]1, HI2, parenchymal hematoma [PH]1, PH2).^12^ The majority of acute hemorrhages were noted on noncontrast head CT; however, MRI alone was used in a minority of cases. High interrater reliability for hemorrhage severity has been previously demonstrated (к = 0.76).^13^

### Sample Size Calculation and Statistical Analysis

The sample size for the original retrospective cohort was determined using preliminary data. Given a mean eGFR for individuals with intracerebral hemorrhage (ICH) of 45 mL/min/1.73 m^2^ (standard deviation [SD] 19.5) compared to 52.5 mL/min/1.73 m^2^ (SD 14.2) in those without and a combined symptomatic and asymptomatic hemorrhage rate of 25% we calculated a required sample size of 63 individuals with ICH and 189 without to show primary differences based on eGFR alone. A total of 345 charts were screened given the likelihood many would fail to meet inclusion criteria and an initial analysis was performed to determine the accuracy of our sample size calculation that showed statistically significant results. For the prospective validation cohort, sample size was calculated based on comparison of mean estimated ICH scores from our retrospective cohort showing the required sample size of 36 total individuals to detect a statistically significant difference with 80% power. We chose to recruit a larger overall sample size to guarantee adequate numbers of individuals with brain MRI and complete data.

Univariate analysis was performed using Student's *t*-tests and Chi-square tests. Subsequent multivariable logistic regression, including age, race, sex, and all variables significant in univariate analysis was performed, with HT (combined symptomatic and asymptomatic) as the dependent variable. A secondary analysis was performed investigating factors predictive of symptomatic hemorrhage.

Model validation—a HeRS score was calculated for each individual and compared to actual hemorrhage status using receiver operating curve analysis. The sensitivity, specificity, positive predictive value, and negative predictive value were generated for multiple cutpoints (Table 2).

**TABLE 2:**
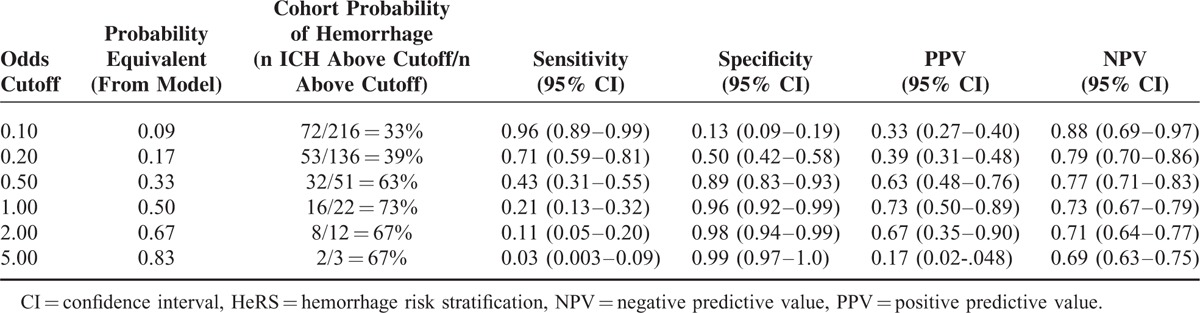
Probabilities of Hemorrhage (Model Vs Cohort), Sensitivity, Specificity, PPV, and NPV for Different Estimated Hemorrhage Odds Cutoffs for the HeRS Score

## RESULTS

### Validation Cohort

A total of 241 patients were included in the present analysis. They were similar in characteristics to our original cohort (Table 3). Eighty-three percent had anticoagulation initiated during their hospital stay. The most common indications were: atrial fibrillation (36%), reduced ejection fraction (11%), and known hypercoagulable state (10%). Consistent with prior findings, in the validation cohort anticoagulation during the hospitalization was not associated with a higher risk of HT (*P* = 0.797); however, being on warfarin at the time of admission was a significant predictor (*P* = 0.002). The average length of stay was 9.8 days (median 7 days) and the majority (n = 64/75) of hemorrhages occurred within the 1st week (mean 4.6 days from admission, median 1 day). Higher peak aPTTr (*P* = 0.660) and INR (*P* = 0.703) values were not associated with increased risk. Results for all variables of interest in univariate analysis are outlined in Table 3. Along with warfarin use on admission, worsening eGFR category (*P* = 0.043), larger infarct volume (*P* < 0.001), higher serum glucose (*P* < 0.001), higher NIH Stroke Scale score (*P* < 0.001), treatment with tPA (*P* = 0.005), hemicraniectomy (*P* = 0.017), and elevated white blood cell count (*P* < 0.001) were associated with higher risk of HT.

**TABLE 3:**
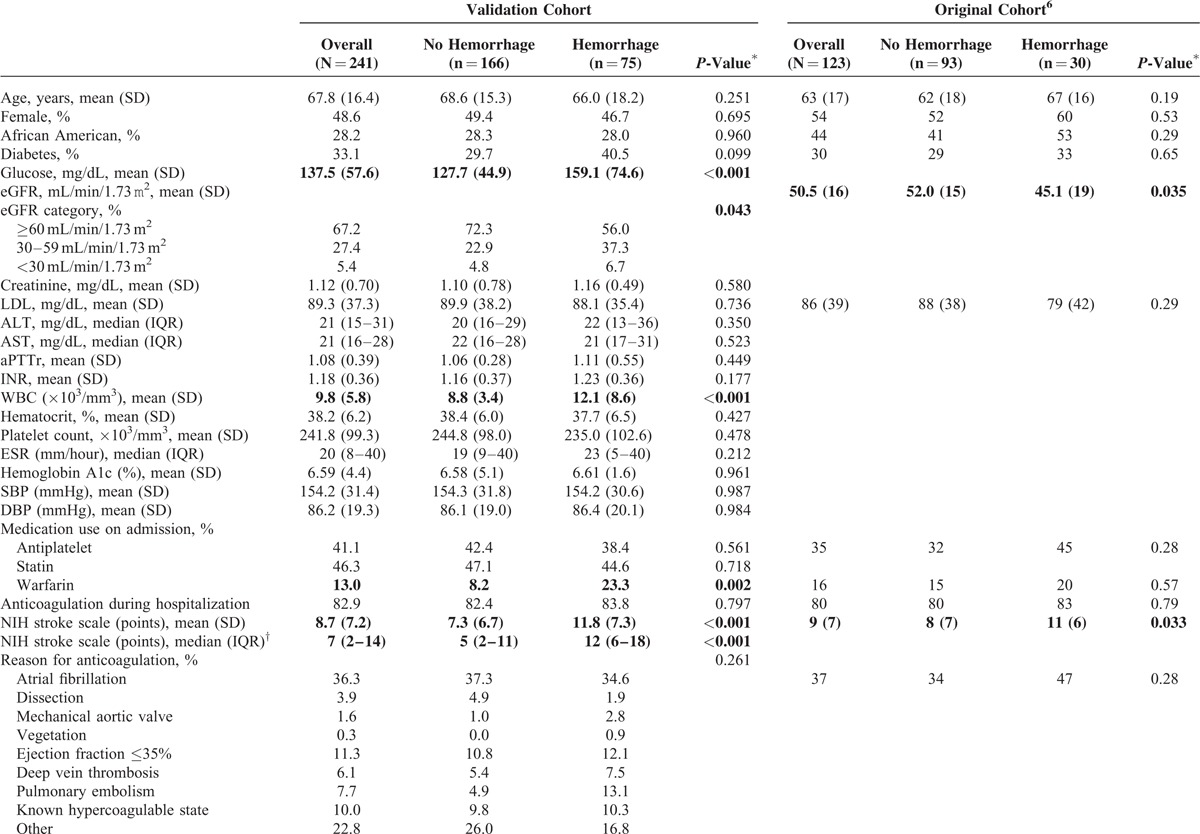
Patient Characteristics by Hemorrhage Status for the Original and Validation Cohorts

**TABLE 3 (Continued):**
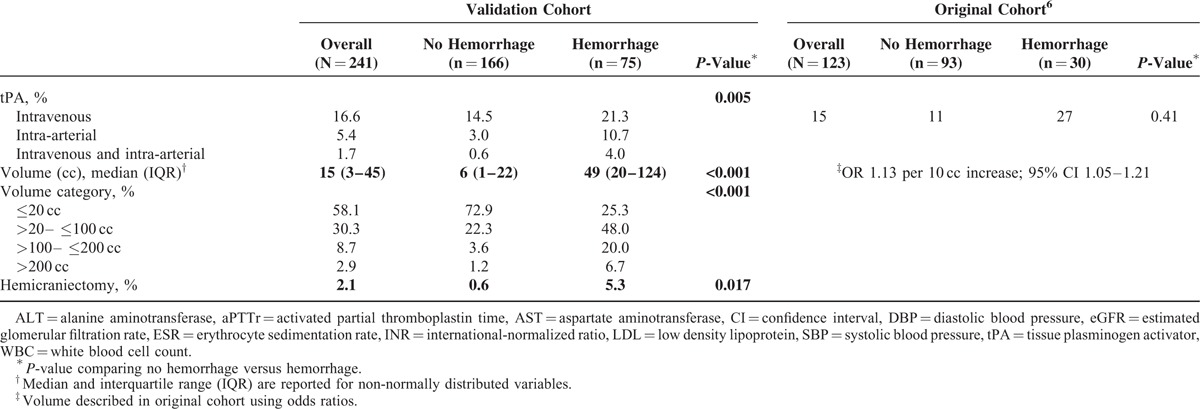
Patient Characteristics by Hemorrhage Status for the Original and Validation Cohorts

In multivariable modeling, renal function and infarct volume remained statistically significant predictors of HT; as well as serum glucose, white blood cell count, and warfarin use prior to admission (which had not been evaluated previously^6^). Intraarterial lysis and hemicraniectomy were also significant predictors in this cohort; however, further sensitivity analyses excluding these patients did not impact results.

### Validation of the HeRS Score

**FIGURE 1:**
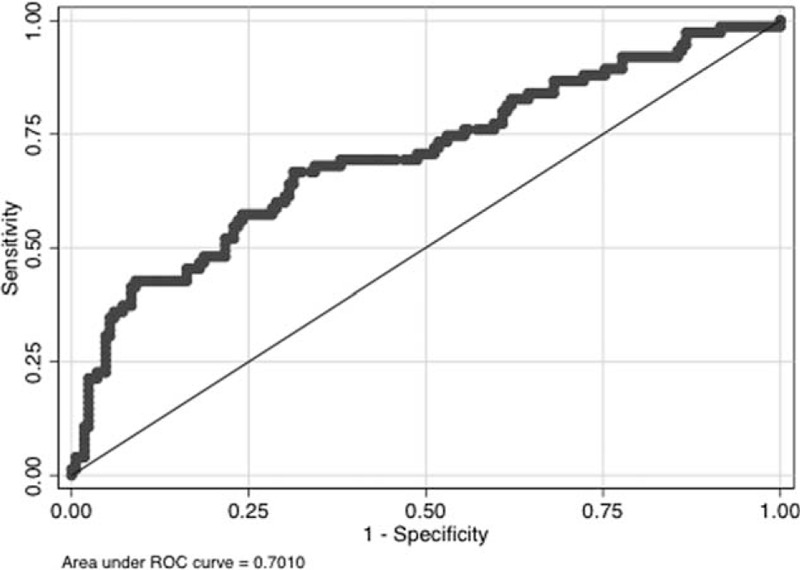
ROC curve for estimated hemorrhage based on the HeRS score versus observed hemorrhage. HeRS = hemorrhage risk stratification, ROC = receiver operator characteristics.

In the validation cohort, the area under the curve (AUC) comparing predicted odds of hemorrhage (HeRS score) to actual hemorrhage status was 0.701 (Figure 1). A secondary analysis incorporating warfarin use on admission, serum glucose, and white blood cell count improved the AUC to 0.854.

### Symptomatic Hemorrhages

Nineteen patients experienced symptomatic HT of their infarct. As the numbers were relatively small, there was no difference in HeRS scores for those with symptomatic versus asymptomatic hemorrhage (*P* = 0.81) and the ability to draw conclusions is somewhat limited; however, patients with symptomatic hemorrhage were more likely to be on warfarin on admission (*P* = 0.024) and in general had higher levels of anticoagulation while in the hospital (mean pTTr values of 1.39 vs 1.02 in those with asymptomatic ICH; *P* = 0.015). When classifying hemorrhages by severity (HI1&2 vs PH1&2), there was also no difference in the average HeRS score (*P* = 0.65); however, patients with more severe hemorrhages were also more likely to have higher pTTr and INR values, and tended to have larger infarct volumes ([mean 91.3 vs 74.2 cc], not statistically significant [*P* = 0.391]). These results are outlined in Table 4.

**TABLE 4:**
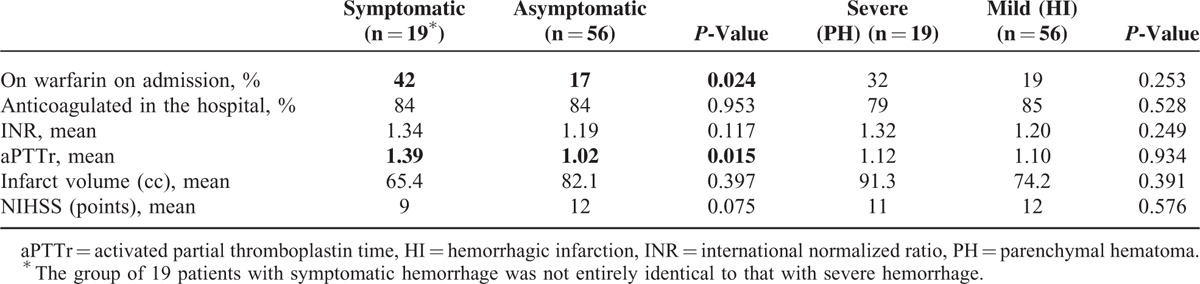
Hemorrhages Classified by Severity.^6^

## DISCUSSION

Following acute stroke, the cerebral vasculature is friable, increasing risk of HT. In patients on anticoagulation, even an initially asymptomatic bleed can ultimately result in significant complications, increasing hospital length of stay, and leading to poorer long-term outcomes and higher healthcare costs.^14^ Thus, the ability to predict any HT when considering anticoagulation is important. A quantifiable risk estimate such as the HeRS score allows for informed decision-making regarding the appropriate intensity of monitoring to detect HT, particularly in institutions without readily accessible neuroimaging. Further, in some settings the score may influence the agent chosen (a heparin infusion that can be rapidly discontinued vs oral therapy with a longer half-life), and provide a tangible risk estimate of potential complications when counseling families.

### Validation of the Hemorrhage Risk Score

We have previously shown that age, infarct volume, and renal impairment (even mild) are predictors of HT. In this study, we validate the HeRS score (based on these variables) in an independent prospectively collected inpatient cohort. Of the 3 risk factors, infarct volume is the most intuitive and has a significant basis in the literature.^15^ The association with age is also consistent with previous studies showing that individuals over the age of 80 who receive intravenous tPA may be at increased bleeding risk.^15^ The effect of renal impairment on bleeding is becoming more apparent.^6,13,16^ Several small studies have also shown that patients with renal failure are more likely to experience intracerebral hemorrhage and poor clinical outcomes.^17–19^ “Uremic platelets” are described in end-stage renal disease,^20,21^ though not in milder renal impairment. Renal failure has also been associated with inflammation that may damage the small vessels, leading to increased permeability of the blood brain barrier over time.^22–26^

### Other Significant Variables

In the validation cohort, hyperglycemia and leukocytosis on admission were associated with increased risk of HT, improving the AUC of the HeRS score to predict HT. This will need to be validated in a unique cohort, but is consistent with prior studies. In the literature, hyperglycemia on presentation has been well associated with poor clinical outcomes.^27–29^ Leukocytosis may indicate an upregulated inflammatory state in the setting of ischemia, or a coexisting underlying medical disorder. We suspect that having large strokes with coexisting medical illness may be the greatest predictor of HT.

Although warfarin use on admission was associated with HT, anticoagulation while hospitalized did not significantly increase risk. This may reflect that the majority of our patients were anticoagulated, yielding low power to detect an effect. Marsh et al^6^ and Flaherty et al^30^ have previously reported a trend toward more severe, symptomatic hemorrhages in those who are anticoagulated, which we did not see in this cohort, likely also because the majority of both symptomatic and asymptomatic hemorrhages were on anticoagulation. However, we did find that a more intense degree of anticoagulation (higher INR and pTTr values) was more likely to result in a hemorrhage that was symptomatic and more severe. Therefore, an alternative explanation is that anticoagulation does not necessarily cause bleeding, but rather increases the likelihood that the hemorrhage will be clinically evident. Interestingly, in the literature, warfarin has been associated with larger volumes and higher rates of expansion of intracerebral hemorrhage irrespective of INR and aPTTr values.^28^ One explanation consistent with both findings is that those presenting on warfarin tend to have atrial fibrillation as their indication for anticoagulation, and atrial fibrillation is associated with larger infarct volumes.^31^ Indeed, patients presenting on warfarin had, on average, 26 cc larger infarcts than those not on warfarin at admission (*P* = 0.023). We did not find that larger infarct volumes or higher NIHSS scores were associated with increased likelihood of symptomatic or severe hemorrhage; however, the averages were quite high for both groups and given the small sample size it is likely that we were not adequately powered to find such a difference within the group who bled.

Our study has several limitations. Because of the high acuity of our patient population, not all were able to undergo MRI, and volume measurement is not as precise using CT due to less discrete borders of ischemia. Additionally, we chose to follow patients only to hospital discharge. However, our purpose was to design a tool to aid in clinical management in the acute setting, and the highest rate of HT occurs in the days immediately following stroke.^2–4^ Finally, we caution that our reported predictive values apply only to our particular cohort—a population likely at high overall risk of HT given the number of medical comorbidities and cardioembolic strokes. Our score would likely have different predictive value in other cohorts, though the sensitivities and specificities would remain unchanged.

Importantly, we chose to include asymptomatic hemorrhages in our primary outcome measure. According to European Cooperative Acute Stroke Study, only severe hemorrhages (PH2) were shown to affect clinical outcome;^32^ however, our prior data suggest that anticoagulation results in larger, more severe hemorrhages.^6^ Therefore, given the potential for worsening of hemorrhage with anticoagulation, we feel it is important to have a reasonable estimate of the risk for all forms of intracerebral bleeding when making decisions regarding clinical management.

Even with these limitations, our data strongly suggest that age, infarct volume, and eGFR are useful predictors of HT. The HeRS score provides a valid calculation of hemorrhage risk for patients with acute ischemic stroke and an indication for anticoagulation. Determination of a specific cut-off point, above which anticoagulation should not be considered, is difficult, as the projected rate of HT is only 1 factor in the decision of whether or not to anticoagulate. We advocate instead that this score be used to help quantify risk for HT in those who must be on an anticoagulant acutely (ie, the patient with a mechanical valve where the daily risk of recurrent embolization is high), and used to guide therapeutic agent of choice, frequency of subsequent monitoring, and how best to council families on complication risk.

## CONCLUSIONS

The HeRS score is a valid predictor of HT of ischemic stroke in patients with an indication for anticoagulation.

## References

[1] Jickling GC Manolescu BN Breaking down barriers to identifying hemorrhagic transformation in ischemic stroke. *Neurology* 2012; 79:1632–1633. 22993281 10.1212/WNL.0b013e31826e9b9d

[2] Khatri P Wechsler LR Broderick JP Intracranial hemorrhage associated with revascularization therapies. *Stroke* 2007; 38:431–440. 17234988 10.1161/01.STR.0000254524.23708.c9

[3] Toni D Fiorelli M Bastianello S Hemorrhagic transformation of brain infarct: predictability in the first 5 hours from stroke onset and influence on clinical outcome. *Neurology* 1996; 46:341–345. 8614491 10.1212/wnl.46.2.341

[4] Hornig CR Dorndorf W Agnoli AL Hemorrhagic cerebral infarction—a prospective study. *Stroke* 1986; 17:179–185. 3515635 10.1161/01.str.17.2.179

[5] Coull BM Williams LS Goldstein LB Anticoagulants and antiplatelet agents in acute ischemic stroke: report of the joint stroke guideline development committee of the American Academy of Neurology and the American Stroke Association (a division of the American Heart Association). *Stroke* 2002; 33:1934–1942. 12105379 10.1161/01.str.0000028456.18614.93

[6] Marsh EB Llinas RH Hillis AE Hemorrhagic transformation in patients with acute ischemic stroke and an indication for anticoagulation. *Eur J Neurol* 2013; 20:962–967. 23521544 10.1111/ene.12126 PMC3711260

[7] Pisters R Lane DA Nieuwlaat R A novel user-friendly score (HAS-BLED) to assess one-year risk of major bleeding in atrial fibrillation patients: the Euro Heart Survey. *Chest* 2010; 138:1093–1100. 20299623 10.1378/chest.10-0134

[8] Gage BF Yan Y Milligan PE Clinical classification schemes for predicting hemorrhage: results from the National Registry of Atrial Fibrillation. *Am Heart J* 2006; 151:713–719. 16504638 10.1016/j.ahj.2005.04.017

[9] Levey AS Bosch JP Lewis JB A more accurate method to estimate glomerular filtration rate from serum creatinine: a new prediction equation. Modification of Diet in Renal Disease study group. *Ann Inter Med* 1999; 130:461–470. 10.7326/0003-4819-130-6-199903160-00002 10075613

[10] Kleinman JT Hillis AE Jordan LC ABC/2: estimating intracerebral haemorrhage volume and total brain volume, and predicting outcome in children. *Dev Med Child Neurol* 2011; 53:281–284. 20875043 10.1111/j.1469-8749.2010.03798.x PMC3026070

[11] The National Institute of Neurological Disorders and Stroke rt-PA stroke study group. Tissue plasminogen activator for acute ischemic stroke. *N Engl J Med* 1995; 333:1581–1587. 7477192 10.1056/NEJM199512143332401

[12] Larrue V von Kummer R Muller A Risk factors for severe hemorrhagic transformation in ischemic stroke patients treated with recombinant tissue plasminogen activator. a secondary analysis of the European-Austrailian Acute Stroke Study (ECASS II). *Stroke* 2001; 32:438–441. 11157179 10.1161/01.str.32.2.438

[13] Marsh EB Gottesman RF Hillis AE Serum creatinine may indicate risk of symptomatic intracranial hemorrhage after iv tpa. *Medicine* 2013; 92:317–323. 24145699 10.1097/MD.0000000000000006 PMC4442012

[14] Boccuzzi SJ Martin J Stephenson J Retrospective study of total healthcare costs associated with chronic nonvalvular atrial fibrillation and the occurrence of a first transient ischemic attack, stroke, or major bleed. *Curr Med Res Opin* 2009; 25:2853–2864. 19916729 10.1185/03007990903196422

[15] Longstreth WT Jr Katz R Tirschwell DL Intravenous tissue plasminogen activator and stroke in the elderly. *Am J Emerg Med* 2010; 28:359–363. 20223397 10.1016/j.ajem.2009.01.025 PMC2837849

[16] Olesen JB Lip GYH Kamper A-L Stroke and bleeding in atrial fibrillation with chronic kidney disease. *New England Journal of Medicine* 2012; 367:625–635. 22894575 10.1056/NEJMoa1105594

[17] Bos MJ Koudstaal PJ Hofman A Decreased glomerular filtration rate is a risk factor for hemorrhagic but not for ischemic stroke: the Rotterdam study. *Stroke* 2007; 38:3127–3132. 17962600 10.1161/STROKEAHA.107.489807

[18] Iseki K Kinjo K Kimura Y Evidence for high risk of cerebral hemorrhage in chronic dialysis patients. *Kidney Int* 1993; 44:1086–1090. 8264139 10.1038/ki.1993.352

[19] Molshatzki N Orion D Tsabari R Chronic kidney disease in patients with acute intracerebral hemorrhage: association with large hematoma volume and poor outcome. *Cerebrovasc Dis* 2011; 31:271–277. 21178352 10.1159/000322155

[20] Di Minno G Martinez J McKean M Platelet dysfunction in uremia: multifaceted defect partially corrected by dialysis. *Am J Med* 1985; 79:552–559. 3933340 10.1016/0002-9343(85)90051-8

[21] Boccardo P Remuzzi G Galbusera M Platelet dysfunction in renal failure. *Semin Thromb Hemost* 2004; 30:579–589. 15497100 10.1055/s-2004-835678

[22] Ford MJ Innes JA Parrish FM The significance of gross elevations of the erythrocyte sedimentation rate in a general medical unit. *Eur J Clin Invest* 1979; 9:191–194. 113219 10.1111/j.1365-2362.1979.tb00922.x

[23] Brouillard M Reade R Boulanger E Erythrocyte sedimentation rate, an underestimated tool in chronic renal failure. *Nephrol Dial Transplant* 1996; 11:2244–2247. 8941585 10.1093/oxfordjournals.ndt.a027143

[24] Yang Y Rosenberg GA Blood-brain barrier breakdown in acute and chronic cerebrovascular disease. *Stroke* 2011; 42:3323–3328. 21940972 10.1161/STROKEAHA.110.608257 PMC3584169

[25] Hawkins CP Mackenzie F Tofts P Patterns of blood-brain barrier breakdown in inflammatory demyelination. *Brain* 1991; 114:801–810. 2043950 10.1093/brain/114.2.801

[26] Marsh EB Gottesman RF Hillis AE Predicting symptomatic intracerebral hemorrhage versus lacunar disease in patients with longstanding hypertension. *Stroke* 2014; 45:1679–1683. 24811338 10.1161/STROKEAHA.114.005331 PMC4442011

[27] Peppe AY Majumdar SR Jeerakathil T The Canadian Alteplase for Stroke Effectiveness Study (CASES) investigators. Admission hyperglycemia predicts a worse outcome in stroke patients treated with intravenous thrombolysis. *Diabetes Care* 2009; 32:617–622. 19131465 10.2337/dc08-1754 PMC2660481

[28] Flibotte JJ Hagan N O’Donnell J Warfarin, hematoma expansion, and outcome of intracerebral hemorrhage. *Neurology* 2004; 63:1059–1064. 15452298 10.1212/01.wnl.0000138428.40673.83

[29] Bruno A Durkalski VL Hall CE The Stroke Hyperglycemia Insulin Network Effort (SHINE) trial protocol: a randomized, blinded, efficacy trial of standard vs. intensive hyperglycemia management in acute stroke. *Int J Stroke* 2014; 9:246–251. 23506245 10.1111/ijs.12045 PMC3904437

[30] Flaherty ML Tao H Haverbusch M Warfarin use leads to larger intracerebral hematomas. *Neurology* 2008; 71:1084–1089. 18824672 10.1212/01.wnl.0000326895.58992.27 PMC2668872

[31] Penado S Cano M Acha O Stroke severity in patients with atrial fibrillation. *Am J Med* 2002; 112:572–574. 12015250 10.1016/s0002-9343(02)01063-x

[32] Fiorelli M Bastianello S von Kummer R Hemorrhagic Transformation within 36 hours of a cerebral infarct: Relationships with early clinical deterioration and 3-month outcome in the European Cooperative Acute Stroke Study I (ECASS I) Cohort. *Stroke* 1999; 30:2280–2284. 10548658 10.1161/01.str.30.11.2280

